# MRI for Lung Cancer Management: Any Closer to Clinical Application?

**DOI:** 10.1002/jmri.70308

**Published:** 2026-04-03

**Authors:** Juergen Biederer, Liisa L. Bergmann, Jeanne B. Ackman, Bruno Hochhegger, Lea Azour, Simon M. F. Triphan, Julien Dinkel, Yoshiharu Ohno, Yoshiyuki Ozawa, Edwin J. R. van Beek, Lena Wucherpfennig

**Affiliations:** ^1^ Diagnostic and Interventional Radiology University Hospital Heidelberg Germany; ^2^ Translational Lung Research Center Heidelberg (TLRC), Member of the German Center for Lung Research (DZL) Heidelberg Germany; ^3^ Faculty of Medicine and Live Sciences University of Latvia Riga Latvia; ^4^ Faculty of Medicine Christian‐Albrechts‐Universität zu Kiel Kiel Germany; ^5^ Department of Radiology, Cardiothoracic Imaging Section Medical College of Wisconsin, Froedtert Hospital Milwaukee Wisconsin USA; ^6^ Department of Radiology, Division of Thoracic Imaging and Intervention Harvard University, Massachusetts General Hospital Boston Massachusetts USA; ^7^ Department of Radiology University of Florida Gainesville Florida USA; ^8^ Department of Radiological Sciences David Geffen School of Medicine at UCLA Los Angeles California USA; ^9^ Department of Radiology University Hospital Nice Nice France; ^10^ Department of Diagnostic Radiology Fujita Health University School of Medicine Toyoake Japan; ^11^ Joint Research Laboratory of Advanced Medical Imaging and Artificial Intelligence Fujita Health University School of Medicine Toyoake Japan; ^12^ Institute for Neuroscience and Cardiovascular Research, Edinburgh Imaging University of Edinburgh Edinburgh UK

**Keywords:** lung cancer, magnetic resonance imaging (MRI), prediction of prognosis, screening, staging, surveillance

## Abstract

Management of lung cancer (LC) encompasses screening, diagnosis, staging, radiotherapy planning and guidance, therapy monitoring and surveillance. Across these domains, magnetic resonance imaging (MRI) offers a range of morphological and functional imaging capabilities—including diffusion‐weighted imaging (DWI), dynamic contrast‐enhanced (DCE) imaging, and whole‐body MRI—to complement established imaging modalities. Recent technical advances have substantially improved the feasibility of lung MRI, enabling more reliable image acquisition and lesion assessment under controlled conditions. In LC screening, meta‐analyses and prospective studies indicate that MRI can detect solid pulmonary nodules above clinically actionable size thresholds with moderate to high sensitivity and a low false‐positive rate. However, the available evidence is largely derived from pilot studies, selected cohorts, and modeling‐based analyses. MRI should therefore be regarded as technically feasible for screening but not yet a validated alternative to low‐dose computed tomography in population‐based programs. For staging, whole‐body MRI incorporating DWI has demonstrated comparable diagnostic performance to standard multimodality pathways in prospective and randomized studies, with potential advantages including reduced radiation exposure and streamlined imaging workflows. In radiotherapy planning, DCE, DWI, and motion‐resolved MRI techniques can improve target delineation and treatment adaptation, but their use remains largely confined to specialized centers. MRI shows promise for therapy response assessment and prognostication through quantitative DCE‐ and DWI‐derived biomarkers, although reported parameters remain heterogeneous and insufficiently standardized for routine clinical decision‐making. Overall, MRI has established clinical utility in selected aspects of LC management, while broader adoption is currently limited by availability, standardization, and validation gaps. Further technical refinement and large‐scale prospective trials are required to define its role in routine clinical practice.

**Level of Evidence:**

5.

**Technical Efficacy:**

Stage 2.

## 1 Introduction

Lung cancer (LC) is worldwide the most common cause of cancer death, owing to the typically late diagnosis and poor prognosis related to a frequently advanced stage of the disease, once symptomatic [1]. The socio‐economic consequences are immense and intense research activities have been undertaken worldwide to improve prevention, early detection, diagnosis and therapy of LC. In all these activities, imaging plays a pivotal role. Besides the current standards, computed tomography (CT) and positron emission tomography (PET) [2], magnetic resonance imaging (MRI) offers unique imaging capacities and can be considered an alternative or valuable adjunct to the standard approaches at all levels from screening to diagnosis, staging, therapy and surveillance. This review synthesizes peer‐reviewed literature published between 2000 and 2025, focusing on meta‐analyses, prospective studies, randomized trials, and large multicenter cohorts when available. Key selection criteria included relevance to lung MRI across screening, staging, radiotherapy planning, and therapy monitoring, with emphasis on studies providing quantitative performance metrics and clinical validation. The article will provide an update on the diagnostic scope, current practice and potential future role of MRI for the management of LC in all domains from early detection (LC screening [LCS]) to lung lesion characterization, LC staging and prognosis, LC therapy and surveillance.

## 2 MRI in Lung Cancer Screening

### 2.1 Nodule Detection

In imaging‐based early detection of LC, that is, LCS, MRI typically plays a minor role. Low‐dose, noncontrast enhanced CT (LDCT) is widely accepted as the workhorse for LCS. As a first line screening modality, LDCT is valued for its speed, robustness and cost‐effectiveness with a high sensitivity and specificity for the detection of malignant lung lesions [3]. In contrast to this, the potential of lung MRI as a first line screening modality is underestimated. One concern is related to the lower spatial resolution of MRI relative to CT. While the latest clinical CT scanners may reach 0.15 × 0.15 mm^2^ at a matrix of 1024 × 1024 in plane and 0.20 mm through plane resolution, the highest spatial resolution of ultrashort echo time (UTE) lung MRI is around 1 × 1 × 1 mm^3^ [4, 5, 6]. Moreover, longer acquisition times in the range of up to 20s for a breath hold make lung MRI susceptible to motion artifacts [7]. However, this small difference in spatial resolution between modalities should not be of practical significance for clinically relevant LCs.

A recent meta‐analysis of 10 studies on the diagnostic performance of MRI for the detection of solid pulmonary nodules revealed a sensitivity of 0.805 (95% CI 0.715–0.871) for nodule sizes of 4–8 mm mean diameter and of 0.985 (95% CI 0.904–0.998) for nodules larger than 8 mm mean diameter, with a pooled average number of only 0.124 false positive nodules per participant [8]. From this, a diameter of 4–5 mm appears to be a realistic detection threshold size for lung nodules with MRI. The meta‐analysis included only three studies using a thin section thickness (< 1.25 mm) and a radial motion‐corrected acquisition. Two of 10 studies used UTE sequences, while most of the other studies (7 of 10) employed different imaging techniques with slice thicknesses of 3–6 mm [8]. The most frequently used approach for the studies included in the meta‐analysis was breath‐hold, three‐dimensional T1‐weighted gradient echo (GRE) sequences with a Cartesian acquisition and different acceleration techniques including volumetric interpolation and parallel imaging [8]. This is consistent with suggested protocols for MRI‐based LC screening [7]. Studies using T2‐weighted short tau inversion recovery (STIR) sequences and T1/T2‐weighted balanced steady‐state free precession acquisitions were included as well. All included studies used either 1.5‐ or 3.0‐T MRI scanners and performed a combination of MRI sequences.

Most current guidelines for LC screening with CT consider only solid lesions with diameters of 6 mm or more (subsolid lesions of 30 mm or more) as clinically relevant (“actionable nodules”) [3, 8, 9]. Nodules smaller than 6 mm are classified as “micronodules,” a term which implicates that these are considered typically irrelevant [10]. Several MRI studies employing CT as the reference standard identified nearly all actionable nodules [5, 11, 12, 13, 14]. In a small study with adjunctive MRI for positive CT screening results, MRI was found to be particularly more efficient in detecting malignant lesions compared to benign, but at a lower overall sensitivity for nodule detection compared to CT [15]. Given the low false positive rate demonstrated with MRI screening, use of MRI as first‐line screening modality could serve to minimize over‐diagnosis and improve the cost‐effectiveness of LCS. In a previous Markov model simulation of LCS in 2019, MRI had a near‐equivalent life expectancy benefit and superior cost‐effectiveness compared to LDCT in the national lung screening trial [16]. The suggestion that MRI could serve as a first‐line screening modality is intriguing but rather speculative. However, both MRI and CT technology, as well as the screening concepts of the studies used for the above‐mentioned simulations, have been further developed since then [3, 9]. For MRI, refinements include high‐resolution UTE sequences, motion‐correction strategies, and accelerated acquisition schemes [17]. Similarly, acquisition speed and spatial resolution of CT have been improved, as have artificial intelligence‐based algorithms for nodule detection and management—the discussion of which is beyond the scope of this review [9]. In screening concepts, risk stratification and inclusion criteria have been revised [3]. Given the obvious technical feasibility, the logical next step would be a large prospective population‐based randomized trial evaluating both MRI and LDCT to prove the noninferiority and cost‐effectiveness of MRI‐based LCS. For the sake of cost‐effectiveness and patient safety, MRI as first‐line screening modality would be used without IV contrast injection.

### 2.2 Nodule Characterization

#### 2.2.1 Nodule Tissue Characterization by Signal Intensity and Morphology

**FIGURE 1:**
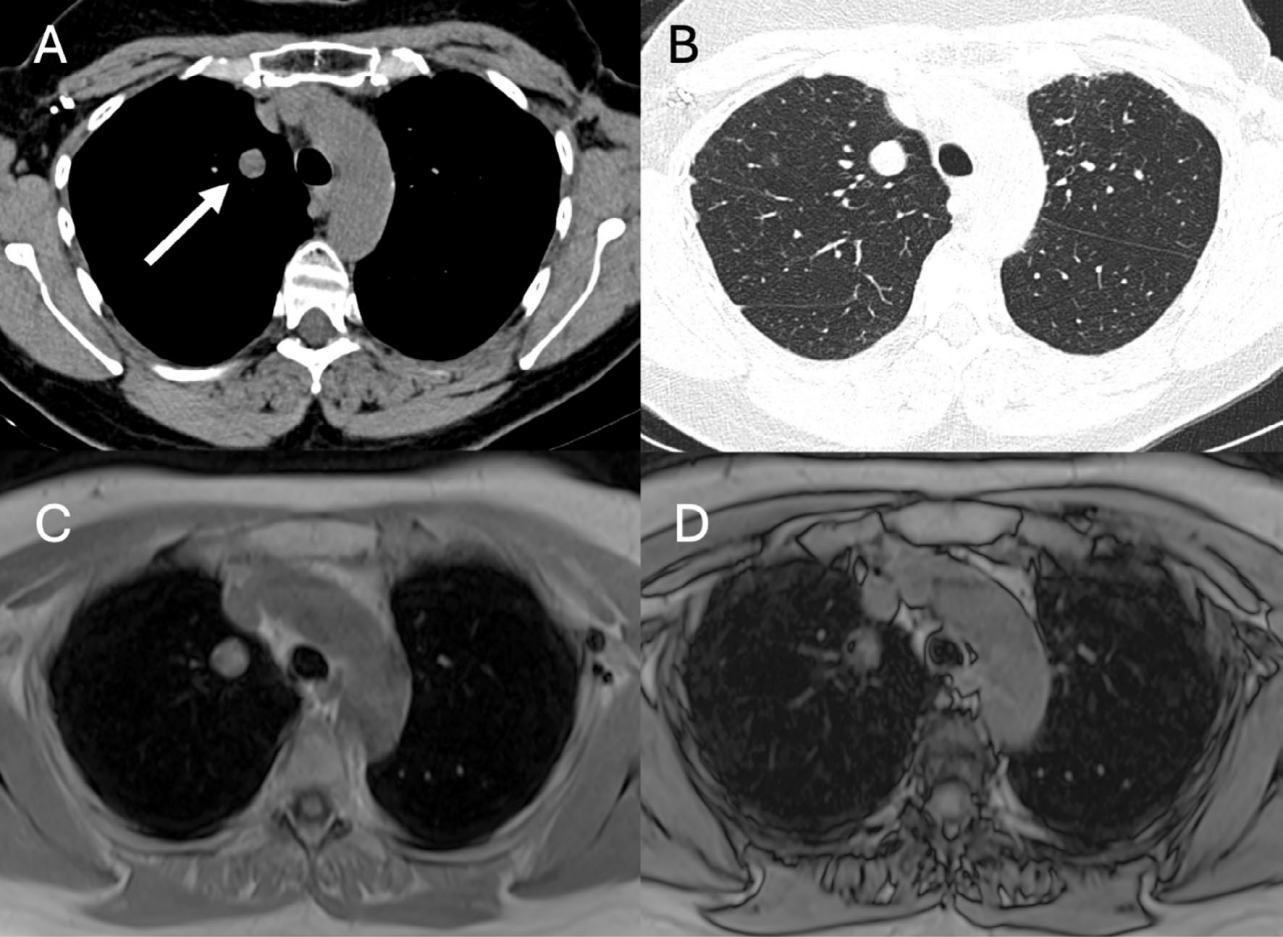
A 59‐year‐old man with incidentally detected pulmonary nodule (arrow). Axial pulmonary (A) and mediastinal (B) CT images show low‐attenuation areas in nodule (−23 HU). T1‐weighted in‐phase (C) and out‐of‐phase (D) MR images show significant focal decrease of signal intensity of a component of the nodule, consistent with intravoxel fat and diagnosis of hamartoma.

In addition to serving as a first line screening modality, MRI might play an interesting role as an adjunct to CT‐based LC screening. The superior tissue characterization properties of MRI, compared to CT, for distinguishing fluid from solid, vascularized, enhancing material frequently allow for a definitive diagnosis of mucoid impaction, cystic lesions, hematoma, abscess, and hamartoma with fatty or cartilaginous content, obviating the need for further intervention in these cases [18, 19, 20] (Figure 1). A case report suggests that a clear delineation of the T2‐hypointense, relatively nonenhancing, curvilinear enfolded visceral pleura amidst homogeneously hyper‐enhancing atelectasis by MRI may confirm the diagnosis of rounded atelectasis [21]. For other solid lesions, noncontrast MRI has only moderate accuracy in distinguishing malignant and benign findings. Carcinoma, metastases, lymphoma, and carcinoid typically show nonspecific low to intermediate signal intensity on T1‐weighted images and a high signal intensity on T2‐weighted images. Imaging with STIR‐sequences for the detection of high signal intensity as a marker for malignant lung nodules has been shown to reach a sensitivity and a specificity of approximately 80% and 60%, respectively [22].

In LCS with CT as well as in clinical practice, the probability of lesion malignancy is typically determined by several factors: (a) lesion attenuation (ground glass, part‐solid, or solid) [23], (b) size, (c) location (central, peripheral, or subplural), (d) morphology (triangular, polygonal, lobulated, spiculated, etc.), and (e) on the assessment of its growth rate [3, 9, 23, 24]. Compared to the CT approach and depending on the MR sequence selection, MRI is considered inferior at characterizing the detailed morphology of a lesion, that is, fine reticulation/spiculation or a ground glass component [12]. UTE sequences appear to be more favorable for this purpose than a Cartesian T1‐weighted three‐dimensional (3D) GRE acquisition with 3–6 mm slice thickness, owing to the higher spatial resolution and the compensation for fast signal decay in the lung with UTEs [5]. In an opportunistic screening setting of a COPD cohort, the sensitivity of the above‐mentioned 3D‐GRE sequence was 17.6% for part‐solid and 13% for ground glass nodules while in a comparable setting, a high‐resolution UTE sequence identified and appropriately classified up to 70% of sub‐solid nodules and 90% of the ground glass opacities [25]. As a result of the lower spatial resolution of MRI even with UTE sequences, the differentiation of small nodules into solid or sub‐solid is difficult with a tendency to categorize sub‐solid as solid lesions rather than the reverse [5].

One of the most detailed approaches for morphologic lesion characterization is the American College of Radiology Lung CT Screening Reporting and Data System (LungRADS) classification [23]. It relies on fixed definitions that include nodules of different densities, pulmonary cysts, and airway nodules, requiring the reporting radiologist to identify all the features that apply and assign the most “representative category” from among a list of about six major categories (e.g., from Category 1 to Category 4X) [3, 23]. Designed for CT interpretation, with the focus on small lesion details, the applicability of this approach to lung MRI appears to be limited. Nevertheless, in a study with high resolution UTE MRI, the agreement for LungRADS classification with CT was excellent (*kappa* = 0.82, *p* < 0.001) [5]. In the opportunistic screening setting of a COPD cohort with standard, non‐UTE sequences of 4 mm slice thickness, the agreement for LungRADS classification from MRI and CT was still high (*κ* = 0.70–1.00), in particular for nodules categorized as having higher risk of malignancy [12].

#### 2.2.2 Nodule Characterization by Assessment of Lesion Growth

Precise assessment of lung lesion growth in follow‐up is a key component of LDCT for LCS. An MRI‐based concept would have to take into account that as a result of partial volume effects and susceptibility artifacts at surfaces between lesions and aerated lung tissue, lesion size measurements on MRI tend to render smaller diameters and volumes than on CT, depending on reconstruction parameters for the CT scan and MR sequences [26]. As expected, the minor (e.g., 0.5–1 mm) underestimation of lesion size on MRI correlates with slice thickness. While lesion sizes were underestimated by 0.5 mm (±1.5 mm) in a study using 3D‐GRE with 4 mm slices, underestimation was only 0.3 mm (±1.7) in a study using thin slice UTE with 1 × 1 × 1 mm^3^ voxel sizes [5, 12]. The slight underestimation of lesion size with MRI may be negligible in most cases, in particular since caliper measurements on CT and MRI are not accurate to 0.5 mm, but it at least partially explains differences to CT when using morphologic classifications such as LungRADS, since a nodule of exactly 8 mm on CT (LungRADS Category 4A, “suspicious”) may appear as just below 8 mm on MRI (LungRADS Category 3, “probably benign”). Besides slice thickness, this slight underestimation is likely explained by the standard practice of measuring lung nodules on lung windows, rather than soft tissue windows. Routinely, a solid nodule, let alone, part‐solid or ground glass nodule, measures slightly more on the CT lung window than on the corresponding soft tissue window [27]. The soft tissue window measurement may therefore more accurately reflect the true size of a solid nodule.

Under the assumption that a slight underestimation of lesion size on MRI is a systematic error, it might be negligible for the assessment of lesion growth in the context of follow‐up examinations. In a longitudinal study with follow‐up of 240 nodules larger than 3 mm in 239 COPD patients, growth detection with MRI after 3 years was in excellent concordance with CT (*κ* = 0.88–1.0), detecting growth of more than 2 mm in 19 nodules and regression of nodule size by more than 2 mm in 24 nodules [28]. Since current recommendations for LDCT screening for LC increasingly emphasize the value of computer‐assisted lung nodule volumetry, it is important that this approach has already been successfully tested in a pilot study with UTE MRI of lung nodules [9, 26].

This comparison to CT regarding morphologic criteria and lesion size assessment has not yet taken into account functional imaging, a particular strength of MRI. Dynamic contrast‐enhanced (DCE) imaging and diffusion‐weighted imaging (DWI), with calculation of the apparent diffusion coefficient (ADC), have been intensively studied for the purpose of detecting malignancy in lung lesions [29, 30].

#### 2.2.3 Nodule Characterization Using DCE Imaging

While MRI as first line screening modality would be applied without intravenous contrast injection, further characterization of suspicious lesions by temporal enhancement characteristics is achieved with DCE and automatic post‐processed subtraction [31]. Internal enhancement generally indicates the presence of vascularity and viable tissue consistent with a malignant or inflammatory (“actionable”) lesion. Cystic or necrotic changes, as well as an abscess or hematoma, may all show peripheral enhancement because of peripheral reactive, inflammatory, or viable tissue [32], but no enhancement in areas of fluid and/or dead tissue. This is similar to cystic or necrotic changes in other anatomy already better characterized with MRI. A study of 36 solitary pulmonary nodules has demonstrated a relevant association between perfusion curve profiles and angiogenesis, with rapid contrast uptake and early washout after 1 min in malignant nodules and gradually increasing enhancement in benign lesions over 3–4 min [31]. Kono et al. had similar results in his study of 202 solitary pulmonary nodules of 1–3 cm in diameter, among which were 31 cases of focal organizing pneumonia, 15 tuberculomas, 12 hamartomas, and 144 primary LCs. Malignant nodules tended to show a more rapid time‐to‐peak enhancement, with benign nodules commonly requiring over 5 min to reach maximal enhancement, but these enhancement patterns overlapped between the two groups [33]. Rather than precisely differentiating malignant from benign solitary pulmonary nodules, DCE MRI is therefore helpful for detecting actionable pulmonary nodules that require further work up, such as malignancy or active inflammation. A lack of contrast enhancement can be interpreted as a strong predictor that a lesion is benign.

Besides this visual, semi‐quantitative approach, mathematical modeling can be applied in order to extract more specific information from DCE perfusion kinetics. Classical pharmacokinetic models (e.g., Tofts model) are used to describe the fractional volumes of bio‐compartments such as the extravascular extracellular space (*V*_e_) and plasma (*V*_p_) and the transfer of contrast media between these compartments (as transfer rate constant *K* ^trans^ from blood plasma to extravascular extracellular space and as transfer rate constant *K*_ep_ back to the blood plasma [34]). Various models have been developed, each with different limitations, for example, calculation of concentration vs. time curves under the assumption of a linear relationship between contrast media concentration and signal intensity, which only applies to a narrow range of contrast material concentrations [35].

In LC imaging, several pilot studies have been conducted using DCE‐MRI with pharmacokinetic modeling and perfusion histogram analyses for the assessment of tumor angiogenesis, therapy‐induced microvascular changes and estimating cancer prognosis [35]. A study of 65 patients (43/65 with lung malignancy) suggests that the ability of *K* ^trans^ and *K*_ep_ to distinguish between benign and malignant lesions reflects differences in angiogenesis [36]. A study of 31 patients (23/31 with lung malignancy) identified *K* ^trans^ and mean ADC from DWI as the best predictors of malignancy among a number of different parameters and concluded that DWI with ADC is more economical, because it does not require intravenous contrast administration [37]. In a group of 93 patients, *K* ^trans^ from DCE MRI was found to differentiate adenocarcinoma (41 patients) from squamous cell carcinoma (29 patients) and small cell LC (23 patients) from non–small cell LC (NSCLC) (70 patients) [38]. In 33 LC patients (15 squamous cell cancers, 12 adenocarcinomas, and 6 small cell cancers), a DCE perfusion histogram and DWI were used to predict LC cell proliferation activity (Ki‐67) in different pathological cancer types [39]. The relationship between quantitative perfusion histogram parameters from DCE‐MRI and the expression of tumor tissue epidermal growth factor receptor (EGFR), vascular endothelial growth factor and EGFR gene mutations has been studied in 44 patients with NSCLC [40].

These examples suggest a high potential for DCE MRI in LC research, but its clinical utility has not yet been confirmed. The available evidence for DCE‐based characterization of lung nodules is largely derived from small, single‐center studies with heterogeneous acquisition protocols, variable quantitative parameters, and suboptimal cross‐vendor standardization. These factors currently constrain reproducibility, comparison across studies, and broad clinical implementation and should be considered when interpreting reported diagnostic and prognostic performance.

#### 2.2.4 Nodule Characterization Using DWI

DWI for differentiating benign from malignant lung lesions relies on the fact that increased cellularity, increased nuclear size and accumulation of macro‐molecular proteins inside cancer result in a restricted diffusion of water molecules and a decreased ADC [30]. The examinations are typically performed at both 1.5 T and 3 T, with 3 T favored for higher signal‐to‐noise ratio but at the cost of increased susceptibility artifacts and motion sensitivity. Acquisition strategies vary widely and include single‐shot echoplanar imaging (EPI), segmented EPI, and fast spin‐echo‐based DWI, using breath‐hold, respiratory‐triggered, or free‐breathing approaches, often combined with parallel imaging or motion correction [41]. DWI protocols most commonly employ low *b*‐values (0–50 s/mm^2^) combined with intermediate to high *b*‐values in the range of 500–800–1000 s/mm^2^, with some studies extending to ≥ 1000 s/mm^2^ for non‐Gaussian diffusion modeling [41, 42, 43]. This substantial heterogeneity in field strength, *b*‐value selection, sequence type, and motion management directly affects ADC estimates, lesion conspicuity, and quantitative reproducibility, contributing to variability in reported diagnostic and prognostic performance and limiting direct comparability across studies [44, 45].

A 2019 meta‐analysis of 37 studies including 4463 lesions (14 studies of DWI including 1116 lesions), of which 69% (*n* = 3090) were malignant, showed a higher diagnostic odds ratio (OR) for DWI than for FDG PET/CT (50 vs. 15, *p* = 0.006) [44]. The median lesion size in the 14 studies of DWI was 22 mm, the typical size category that would usually undergo resection or biopsy [23]. These results were confirmed by a more recent meta‐analysis including 10 studies of DWI with 948 solid lesions showing a sensitivity and specificity of DWI for malignancy of 85% and 91%, respectively (area under the curve [AUC] 0.94, diagnostic OR for DWI higher than for FDG PET/CT [55 vs. 16]) [45].

The potential clinical applications of DWI with ADC mapping are manifold. For example, recent research on lung lesion characterization has shown that a lower ADC and higher T2 contrast ratio may differentiate high‐grade adenocarcinoma from low grade (153 patients with adenocarcinoma; 103 low‐grade) [42]. A lower ADC and higher signal intensity on T1‐weighted and T2‐weighted fast spin echo (FSE) images have been used to distinguish LC from progressive massive fibrosis (latter is typically T2‐hypointense) in a study of 60 patients (30 of whom had LC) [43].

Further research takes advantage of additional effects at the far ends of the signal intensity to *b*‐value relation. ADC is usually calculated from the slope of the almost linear course of the graph at intermediate *b*‐values (“pure” diffusion D). However, at very short *b*‐values pure diffusion (*D*) is overlaid with a perfusion fraction (*f*) resulting in a higher signal intensity than from pure diffusion. This effect is used for intravoxel incoherent motion (IVIM) imaging for analysis of the perfusion component. At the other end of the curve with long *b*‐values, the non‐Gaussian distribution of diffusion due to membranes again contributes to a higher signal than pure diffusion (kurtosis), resulting in a signal increase and a kurtosis‐derived diffusion coefficient (*D*_app_). This effect is used for diffusional kurtosis imaging (DKI), for example, for analysis of the cellularity of a lesion [41]. It is not difficult to deduce that both components, IVIM for the perfusion component and DKI for the cellularity of a tumor, are interesting candidates for the differentiation of malignant and benign lesions.

The clinical use of DWI for LC detection remains limited despite these promising results and despite advantages over DCE including lack of need for intravenous contrast. The lung remains a challenging site for the most widely used form of DWI–EPI. EPI produces image distortions in the presence of B0 field inhomogeneities that exist in the chest, in addition to signal loss due to short T2* GRE sequences. It therefore frequently fails to produce high‐quality acquisitions. The FSE variant of DWI has the potential for less signal loss and distortion. However, alternative approaches like SPLICE or non‐Carr‐Purcell‐Meiboom‐Gill (CPMG) FSE are needed since the introduction of diffusion weighting gradients violates the CPMG condition upon which conventional FSE relies [46]. These approaches may yield additional potential for DWI specifically in the lungs since FSE should be more robust for depicting diffusion in small lesions surrounded by aerated lung parenchyma.

In summary, while DWI‐based characterization of lung nodules has potential [44, 45], the lack of standardized acquisition protocols and quantitative parameters applies similarly, and a broad clinical implementation is not yet realized. As a next step, studies with appropriate design to address these deficits will be needed.

Multi‐parametric imaging including both DCE‐ and diffusion‐weighted MRI for the identification of malignant nodules could play an interesting role as an adjunct to CT providing ad hoc, noninvasive on‐site nodule characterization. Ideally, this means of assessment would comprise part of the prospective population‐based randomized trials for noninferiority of MRI as first line screening modality, as discussed earlier.

## 3 MRI in Lung Cancer Staging

### 3.1 T‐Staging

In LC staging, imaging is used to assess the local tumor size and its relationship to surrounding structures (T‐stage according to the Union for International Cancer Control), lymph node metastases (N‐stage), and distant metastases (M‐stage) [47]. Theoretically, the earlier‐mentioned slight underestimation of lesion size with MRI compared to CT might lead to an under‐staging of the T‐denominator in cases when the longest diameter on CT just matches the criteria of a certain size category, while measurement on MRI just misses the criterion by 1 mm [12]; however, for such small potential size discrepancies, treatment choices are guided by the ensemble of imaging findings, other test results, physical examination findings, and patient symptoms and preferences.

**FIGURE 2:**
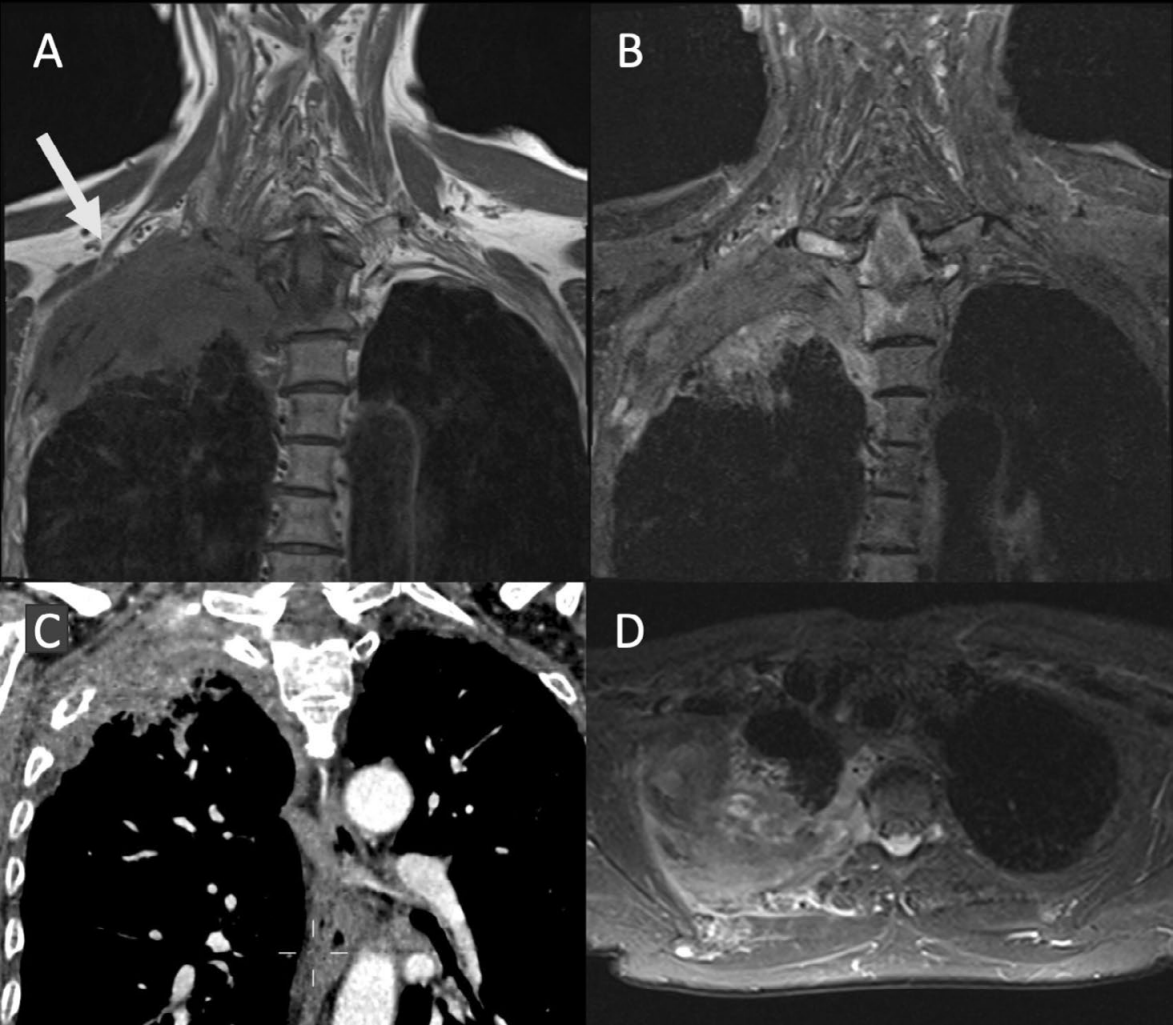
Superior sulcus (Pancoast) tumor in a 62‐year‐old female patient (arrow). Coronal T1‐weighted fast spin echo (A) and STIR images (B) and the axial STIR image (C) clearly show tumor invasion from the right lung apex into the thoracic wall and vertebral column with identification of the brachial plexus and superior soft tissue contrast compared to CT (C).

**FIGURE 3:**
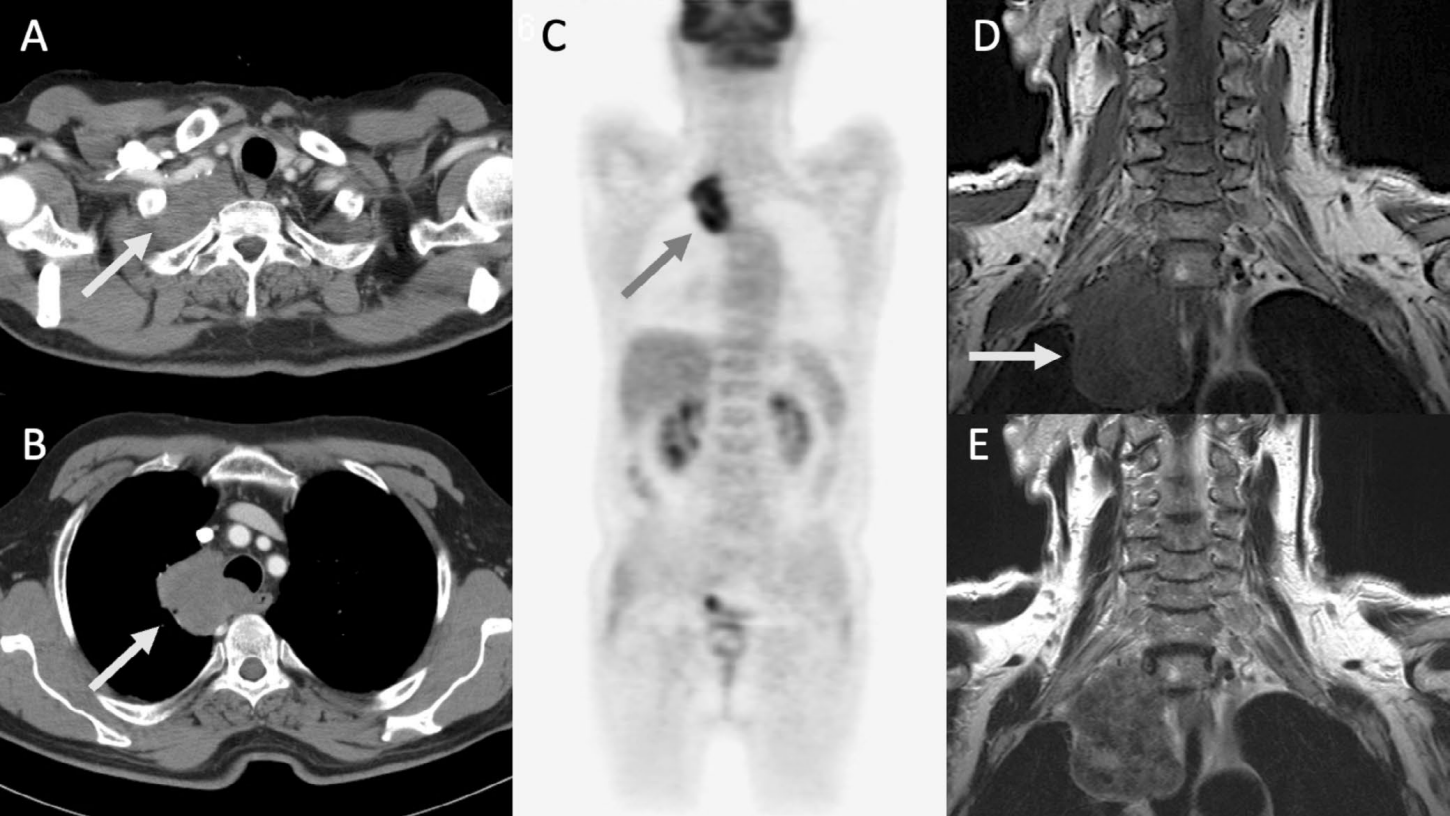
Superior sulcus (Pancoast) tumor (arrow) in a 62‐year‐old male patient. Transverse CT images (A and B) whole body PET (c) and coronal T1‐ (D) and T2‐weighted (E) MRI show tumor invasion from the right lung apex into the thoracic wall but sparing the vertebral column.

**FIGURE 4:**
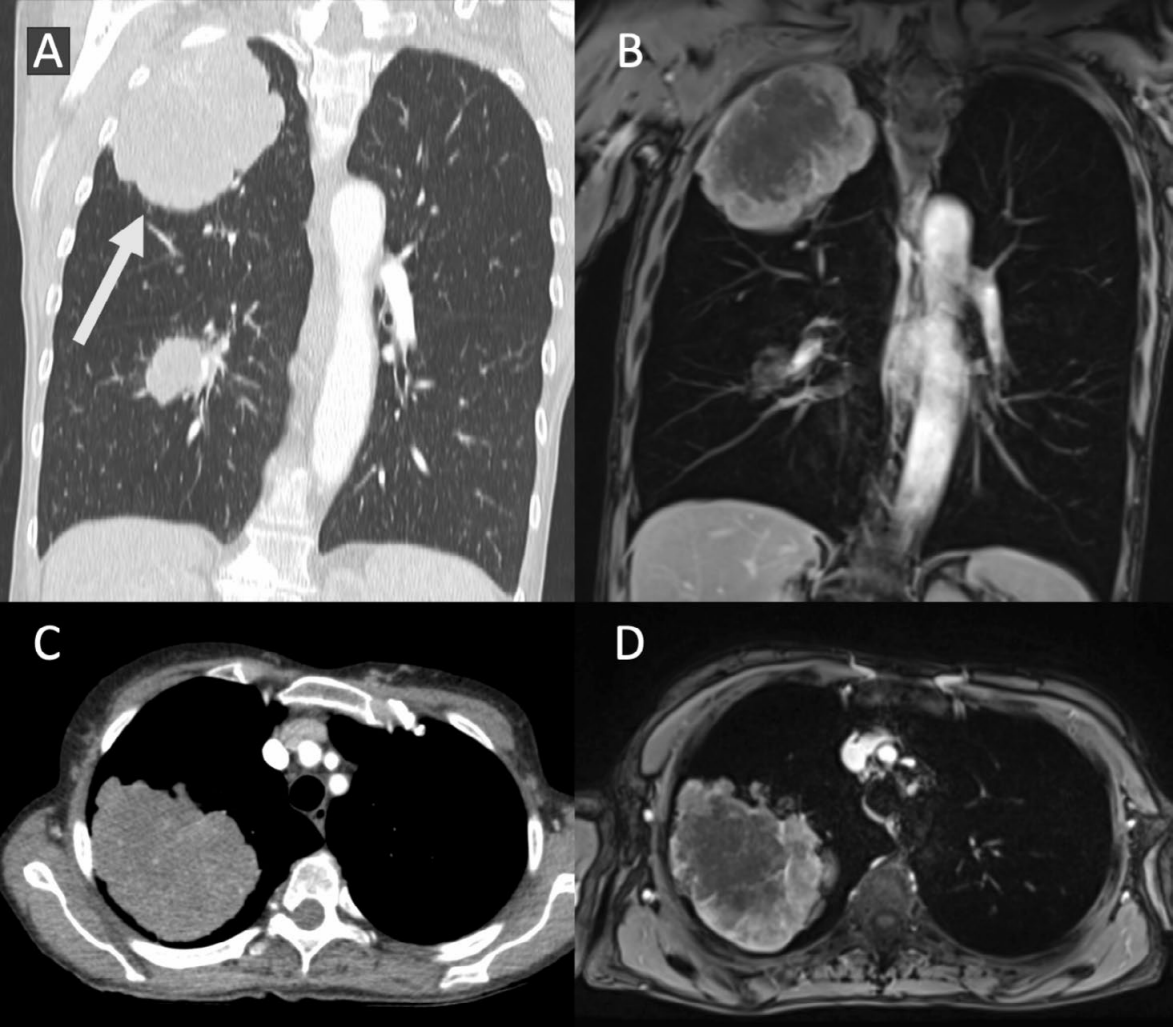
A 63‐year‐old female patient with a mass (arrow) in the right upper lung lobe and suspected lung cancer. Transverse and coronal contrast‐enhanced CT (A and C) was used to plan a CT‐guided biopsy, which produced only necrotic material. A second biopsy after contrast‐enhanced MRI (B and D: contrast‐enhanced 3D‐gradient echo fat‐suppressed T1‐weighted images) allowed for targeted biopsy of vital tissue and revealed a renal cell carcinoma metastasis.

**FIGURE 5:**
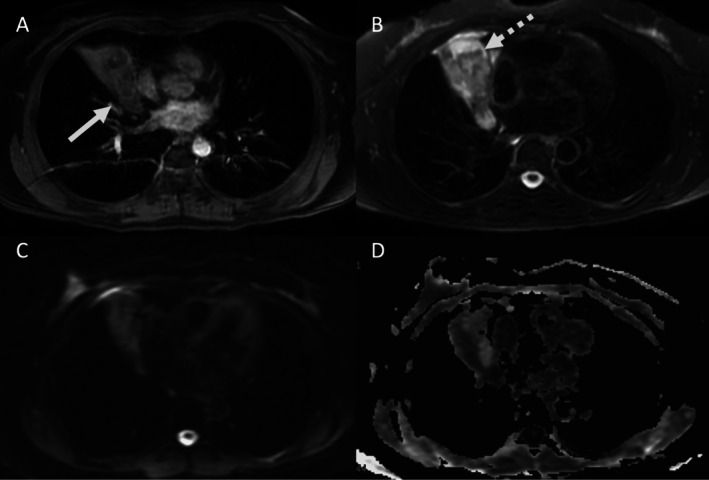
74‐year‐old male patient with pulmonary squamous cell lung cancer. (A) The axial T1‐weighted gradient echo fat sat post contrast series demonstrates a middle lobe mass with 7 cm in maximum diameter and adjacent atelectasis (arrow). (B) The axial T2‐weighted fast spin echo acquisition with fat signal suppression shows a hyperintense middle lobe atelectasis with clear separation from hypointense tumor tissue (dotted arrow). (B) Axial diffusion weighted imaging (*b* = 1000) shows hyperintensity of the complete lesion with distinct decreased intensity on the ADC map (D) in the lateral portion of the lesion.

For very small cancers of the T1a stage (1 cm and smaller), similar considerations apply regarding the sensitivity of MRI for solid, part‐solid, and ground‐glass nodules, as already discussed above for lung nodule detection [5]. Principally—although there are individual exceptions to the rule—a T3 classification confers that surgical resection might be technically possible, whereas a T4 classification suggests an unfavorable situation for resection. A vintage and still useful application of MRI is the evaluation of superior sulcus tumors (Pancoast tumors). Tumors limited to the first and second thoracic nerve roots may still be considered resectable (T3), while involvement of C8 and above as well as invasion into the plexus brachialis, subclavian artery and vein and the vertebral column indicates an unfavorable situation for resection (T4) [48] (Figures 2 and 3). Also, for other tumor locations, invasion into adjacent structures defines surgical options even more than the size of the lesion. The 2025 9th TNM classification denotes invasion into the chest wall, the pericardium and the phrenic nerve as T3 and invasion into the diaphragm, the mediastinum, the carina, the trachea, the heart and great vessels, the esophagus and the recurrent laryngeal nerve as T4 [47]. In the domain of invasiveness detection, MRI excels on account of its higher soft tissue contrast than CT. Invasion into the chest wall and the mediastinum can be better detected and delineated by MRI than on CT [49]. In a study comparing whole‐body MRI (WB‐MRI) at 3 T and PET/CT on 165 LC patients with histopathologic reference, MRI with STIR sequences detected 106/123 cases of chest wall invasion compared to PET/CT, which detected 101/123 [50]. Recent work on hybrid imaging suggests a particularly beneficial combination of MRI and PET with a high predictive value for pleural invasion by small LCs [51, 52]. Post‐contrast subtraction imaging can be applied to reliably indicate the unequivocal presence of enhancement and differentiate it from necrosis or hemorrhage, both of which may be misinterpreted as solid cellular tissue on CT secondary to similar attenuation [32]. This might be particularly helpful for the detection of viable tissue prior to biopsy of large necrotic lesions (Figure 4). The excellent tissue contrast of MRI also contributes to the differentiation of tumor from post‐obstructive atelectasis [53, 54], pneumonitis [55, 56], radiation fibrosis [57], and rounded atelectasis [58] (Figure 5).

### 3.2 N‐Staging

In the staging of mediastinal lymph nodes, surgical resection is usually still favorable in N2 nodes (ipsilateral mediastinal or subcarinal lymph node involvement), but not for N3 nodes (contralateral mediastinal, contralateral hilar or supraclavicular lymph node involvement) [47]. Both CT and MRI are capable of lymph node detection and size measurement. Since lymph node size has unsatisfactory sensitivity and specificity as an indicator of malignancy, clinical practice uses PET and PET/CT for the detection of mediastinal lymph node involvement. However, a prospective study on mediastinal lymph node metastases in 250 patients with NSCLC showed equal or even higher sensitivity, specificity and overall accuracy of MRI with STIR (82.8%/77.4%/86.8%) and MRI with DWI (74.2%/71.0%/84.4%) compared to PET/CT (74.2/69.9%/85.6%) [59]. Another study of 88 NSCLC patients with histopathologic reference found a significantly higher accuracy of MRI with DWI of 0.89 compared to PET/CT (0.78), mainly related to less false positive findings with MRI, in particular in patients with pneumoconiosis [60]. A meta‐analysis from 2016 of 12 studies (1122 patients; 4302 lymph nodes) confirmed a pooled sensitivity and specificity of 0.87 and 0.88, respectively, for the detection of malignant mediastinal lymph node involvement with DWI [61]. Computed DWI has determined *b* = 600 s/mm^2^ as the best *b*‐value for mediastinal N‐staging in 245 NSCLC patients with 114 metastatic and 114 nonmetastatic mediastinal and hilar lymph nodes [62]. In summary, available evidence confirms the feasibility of mediastinal N‐staging with STIR and DWI sequences.

### 3.3 M‐Staging

Development of distant metastases (M1) in LC patients drastically worsens prognosis and indicates a primarily systemic therapy approach [47]. TNM 9 has introduced a sub‐division of M1 (distant metastases) into M1a through M1c2 with slight differences in prognosis. Five‐year survival drops from below 10% for M1a and M1b to 0% for M1c1 and M1c2 [2]. In current clinical staging practice, the standard procedure would be a chest and abdominal pelvic CT (or PET/CT) complemented with a brain MRI. Owing to the high glucose utilization of the brain itself, PET has a low sensitivity for detection of brain metastases, and additional imaging with MRI (alternatively contrast‐enhanced CT) is needed [50]. Given the high sensitivity of MRI for organ, soft tissue and bone lesions including brain metastases, WB‐MRI has been successfully applied as an alternative, one‐stop shop staging approach. Ohno et al. have shown an accuracy of WB‐MRI with DWI of 0.877 comparable to the accuracy of FDG PET/CT (0.882) in 203 NSCLC patients [63]. Yi et al. used 3.0‐T WB‐MRI and reached an accuracy of 0.86 equivalent to FDG PET/CT (0.86) on a study of 165 NSCLC patients [50]. Since then, several studies have confirmed the diagnostic noninferiority of WB‐MRI with DWI, T2‐weighted, and T1‐weighted (pre‐ and post‐intravenous gadolinium contrast agent) imaging to the standard procedures and proven the cost‐effectiveness and time saving capacities of this approach [64, 65]. Consequently, hybrid imaging using whole body PET/MRI is undergoing investigation for staging purposes as well [66]. A potential weakness of this approach is the sufficiently sensitive and specific recognition of stage M1a, which includes separate nodules in the contralateral lung. For these, similar considerations apply regarding the sensitivity of MRI for solid, part‐solid, and ground‐glass nodules, as already discussed above for lung nodule detection [5].

### 3.4 WB‐MRI for Comprehensive LC Staging

The Streamline L trial represents a landmark randomized prospective study evaluating the diagnostic accuracy and clinical efficiency of WB‐MRI compared with conventional multimodality staging in newly diagnosed NSCLC, including PET/CT [65]. Conducted across 16 centers in England, this multicenter trial enrolled 353 patients and provided robust real‐world evidence that WB‐MRI offers comparable sensitivity (50%) and specificity (93%) to standard imaging pathways (54% and 95%, respectively) for detecting metastatic disease. Importantly, WB‐MRI reduced the staging duration by nearly a week (13 vs. 19 days) and lowered the overall diagnostic cost per patient by approximately 50%, without compromising diagnostic confidence or treatment decision accuracy. This trial highlighted the potential of WB‐MRI as a streamlined, radiation‐free alternative to traditional staging approaches that rely on PET‐CT and additional targeted imaging. Despite similar diagnostic accuracy, the efficiency gains and reduced healthcare burden suggest a pivotal role for WB‐MRI in integrated oncologic pathways, particularly in institutions prioritizing rapid, comprehensive, and cost‐effective staging of NSCLC.

**FIGURE 6:**
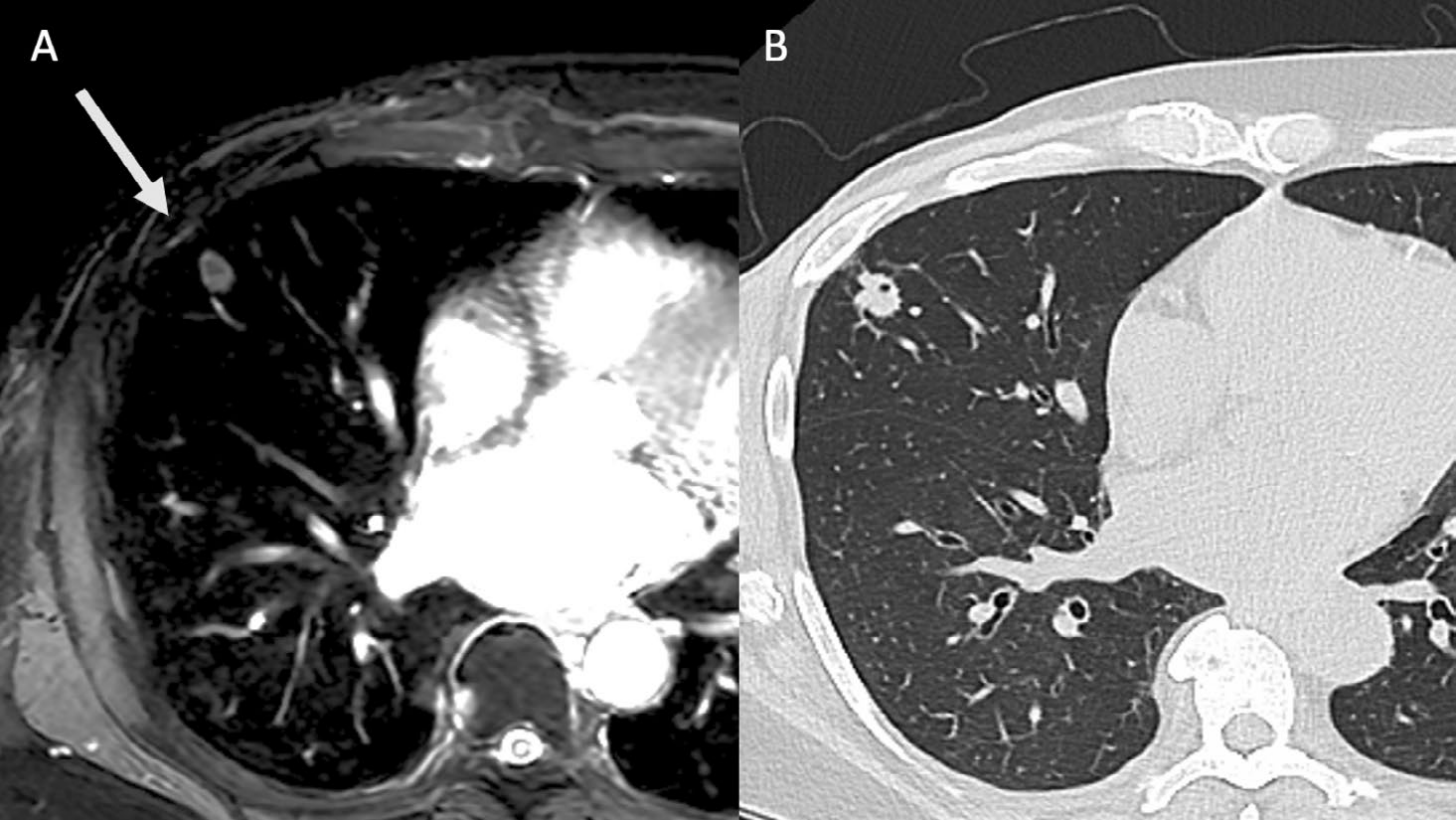
Lung metastasis from a moderately differentiated ductal adenocarcinoma of the pancreas with mucinous differentiation (arrow). (A) The T1/T2‐weighted balanced steady‐state free precession (bSSFP) acquisition (TR 3.61, TE 1.5, TI 200, BW 434.02, 1.5 T field) shows a well‐defined lesion in the lateral segment of the middle lobe. (B) CT image of the same lesion.

Besides LC, pulmonary metastases are a common finding in malignancies, in which 20% of metastatic disease is isolated to the lungs [67] (Figure 6). Since an increasing number of oncological patients routinely undergo regular abdominal MR cancer screening as the current standard of care for staging of extra‐thoracic cancers, the addition of Chest MR to abdominal MR screening as a ‘one‐shot’ study would be similarly effective for detection of pulmonary metastases and could decrease the overall staging time for other malignancies as well. Its use would also help to reduce repeated exposure to ionizing radiation for at‐risk populations, including pregnant patients, children, and patients with radiation‐sensitive genetics.

## 4 MRI in Lung Cancer Therapy

The diagnostic capacities of MRI for detection and staging of LC have also been demonstrated for monitoring response to therapy. Several studies have addressed the evaluation of *early therapy response* with DCE and/or DWI MRI [41, 68, 69]. To sensitively distinguish chemo‐sensitive and chemo‐resistant tumors at the early phase of therapy would allow for earlier treatment adjustment and may prevent continuation of costly regimens proven ineffective.

### 4.1 DCE MRI in LC Therapy

DCE MRI by its principle is the ideal tool for the assessment of tumor vascularity. Pharmacokinetic modeling allows for calculation of semiquantitative parameters such as an increase in capillary permeability, volume of extravascular‐extracellular space, and tumor perfusion [68]. These permit distinction of benign vascularity from malignant vascularity, the latter of which is accompanied by features including spatial heterogeneity and chaotic structure, high permeability to macromolecules, and heterogeneity of vascular density [69]. DCE detection of these features could assist assessment of prognosis and response to treatment, including assessment of early response and overall response during and after therapy.

#### 4.1.1 DCE MRI for the Prediction of Response Prior to Therapy

*Pre‐therapy*, Lee et al. found in a study of 24 NSCLC patients that tumor vascularity and intra‐tumoral heterogeneity in wash‐in contrast kinetics are associated with high tumor metabolism (PET SUV_max_) and discussed that the balance between vascularity and glucose metabolism in a tumor could prove to be an important indicator of its biological status and resistance to treatment [70]. Perfusion and permeability parameters from DCE MRI were found to be predictive of the efficacy of radiochemotherapy in 36 stage III NSCLC patients with the extravascular extracellular space (*V*_e_) of < 0.24 giving the largest area under curve of 0.865 to predict responders [71]. In a later study on 111 stage III NSCLC patients, a high transfer rate constant *K* ^trans^ was found to be predictive for response to immuno‐radiochemotherapy and prolonged progression‐free and overall survival [72].

#### 4.1.2 DCE MRI for the Assessment of Early Therapy Response

Other studies have addressed the predictive value of DCE MRI for the *response to therapy*. In a study on 114 NSCLC patients receiving radiochemotherapy, DCE MRI (i.e., maximum relative enhancement ratio and slope of enhancement) was found to be predictive of the efficacy of treatment [73]. In 98 LC patients receiving tyrosine‐kinase inhibitor or platinum‐based chemotherapy, higher contrast uptake pre‐treatment and early post‐treatment was associated with treatment response and better prognosis [74]. Sequential DCE‐MRI with the transfer rate constant (*K* ^trans^) as biomarker appeared to be useful for early noninvasive therapy assessment in immuno‐chemotherapy of 40 LC patients, but with a coefficient of variation of up to 48.7% in smaller lung lesions [75]. Positive enhancement integral, signal enhancement ratio, and maximum slope of increase in DCE‐MRI were described as indicators of early chemotherapy response in 22 LC patients [76]. For the *early response after radiotherapy*, transfer rate constant (*K* ^trans^) from DCE MRI and tumor volume change detect early effects of treatment at both cohort and individual lesion levels [77].

### 4.2 DWI in LC Therapy

#### 4.2.1 DWI for the Prediction of Response Prior to Therapy

**FIGURE 7:**
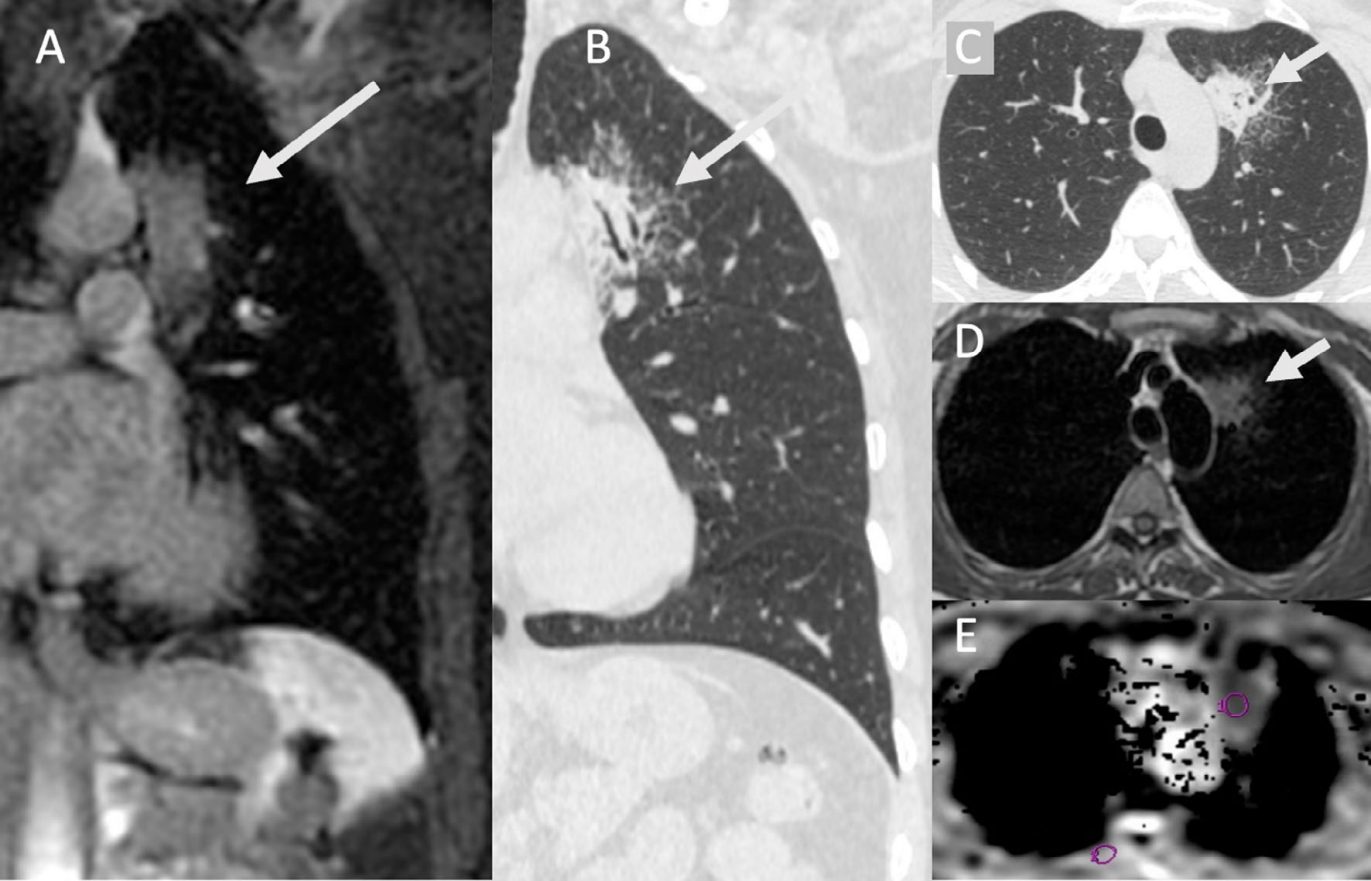
A 42‐year‐old female with a subsolid lesion and consolidation in the juxta‐mediastinal left upper lobe, final diagnosis of adenocarcinoma. A coronal T2‐weighted image with fat signal suppression (A) and a transverse T1‐weighted fast spin echo acquisition (D) show an unspecific, intermediate signal intensity. The ADC map from DWI shows diffusion restriction (E); transverse CT (C) and a coronal reconstruction from CT (B) for reference.

A couple of studies have addressed the predictive value of DWI for the response *prior to therapy* (Figure 7). Ohno et al. have found DWI to be superior to FDG PET/CT for the prediction of tumor response to chemoradiotherapy in 64 patients with stage III NSCLC [78]. A combined model of IVIM imaging and DKI reached an AUC of 0.968 for the detection of responders prior to chemoimmunotherapy in 72 NSCLC patients [79]. In a small study on 15 NSCLC patients scheduled for stereotactic body radiation therapy (SBRT), Iisuka et al. have shown that a low ADC value on DWI and a high SUV_max_ on FDG PET (ideally combined) indicate poor prognosis [80].

There appears to be a tendency toward a good response to standard chemotherapy and radiation therapy in tumors with low pretreatment ADC and good response to vascular disrupting agents with high pretreatment ADC [41, 68]. In addition to DWI, Ohno et al. found amide proton transfer weighted chemical exchange saturation transfer MRI to have similar predictive capacity to DWI and FDG‐PET/CT for the effect of chemoradiotherapy in a study on 84 stage III NSCLC patients [81].

In patients undergoing LC surgery, DWI with ADC mapping has not yet been shown to be helpful for the prediction of outcome. Usuda et al. found DWI with ADC mapping to be not prognostic in 227 patients for resected LC, while SUV_max_ from FDG PET correlated well with the T and N factors [82]. However, more recent work on 65 NSCLC patients suggests that more sophisticated analyses of DWI, including IVIM imaging, may predict tumor differentiation, lymph node status, and pleural invasion prior to surgery [83]. Only recently, Moon et al. have successfully applied a deep learning approach for the analysis of DWI with multiple *b*‐values covering the whole range from very low (IVIM‐effects present) to very high (diffusion kurtosis effects present) to predict survival after resection in 100 NSCLC patients [84].

#### 4.2.2 DWI for the Assessment of Early Therapy Response

**FIGURE 8:**
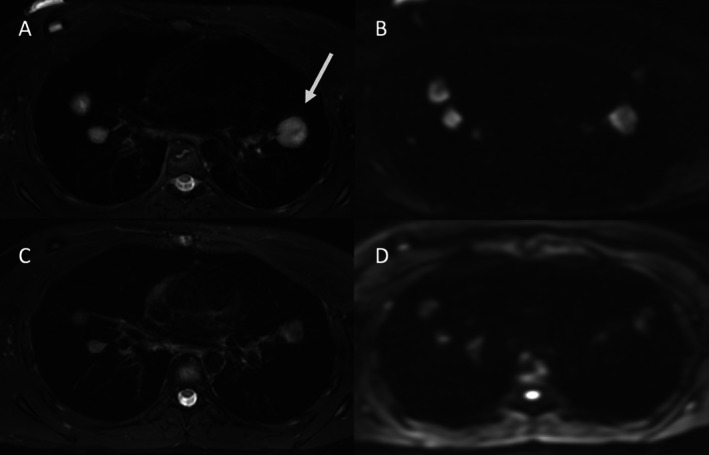
A 52‐year‐old male patient with history of metastatic pulmonary adenocarcinoma and multiple pulmonary metastases. (A) The axial T2‐weighted fast spin echo acquisition with fat signal suppression shows a dominant left upper lobe nodule with a maximum diameter of 28 mm. (B) The axial diffusion weighted image (*b* = 800) shows marked diffusion restriction with hyperintensity of all lung nodules, including the dominant lesion with a respective decreased intensity on ADC map (not shown); (C) The axial T2‐weighted fast spin echo acquisition 3 months after chemotherapy shows a decreased size of all pulmonary nodules with the dominant nodule now measuring 21 mm. (D) The axial diffusion weighted imaging (*b* = 800) shows only a mild, reduced diffusion restriction of the dominant left upper lobe nodule.

Several studies have addressed the evaluation of *early therapy response* with DWI. In a study on 192 NSCLC patients, a high pre‐therapy ADC was associated with worse outcome, while initially low ADC values with increase during and after chemoradiotherapy predicted tumor regression [85]. Zhou et al. found an increase of the ADC after chemotherapy correlated with tumor size reduction in 19 LC patients [86]. In a study on 28 patients (11 SCLC and 17 NSCLC) the reduction in SUV and the increase in ADC correlated well in predicting response to chemotherapy [87]. Higher ADC change rates toward increase were found to correlate with short‐term efficacy after the first course of chemotherapy in 25 NSCLC patients [88]. A higher ADC change rate from the first to the second course of chemotherapy was found to indicate chemo‐sensitivity in 75 NSCLC patients [89]. In 18 stage IV NSCLC patients receiving anti‐angiogenetic chemotherapy an ADC‐change greater than 3 was found to predict longer overall survival [90]. IVIM imaging and ADC have been reported to detect response to chemotherapy in 51 NSCLC patients [91]. In therapy of 20 NSCLC patients with immune checkpoint inhibitors, ADC histogram changes with decreased kurtosis ADC at 4 weeks and increased ADC at 8 weeks on IVIM MRI were associated with objective responses and longer progression‐free survival [92]. In summary, an increase in the ADC with ADC histogram shift toward higher ADC and a decrease in signal intensity on high‐*b*‐value images following treatment with chemotherapy or radiotherapy predict a favorable response and improvement in overall survival and progression‐free survival in NSCLC patients (Figure 8). If there is secondary tumor necrosis, a further increase in the ADC will be observed [41]. However, a very initial decrease in the ADC due to changes in the tumor microenvironment may still be indicative of good response [41].

#### 4.2.3 DWI for the Assessment of Response to Therapy and Surveillance

Similar changes of ADC were observed following radiotherapy in a couple of small studies. A study in 12 NSCLC patients showed that directly after definitive radiotherapy, patients with a good response had higher ADC values than nonresponders [93]. A study on follow‐up after SBRT in 18 NSCLC patients (20 lesions) found only heterogeneous enhancement and heterogeneous T2 signal, and inconclusive results for DCE and ADC, with no single parameter being diagnostic by itself in the four recurrent tumors [94], while another study on 14 patients after SBRT found a significantly lower ADC in the three recurrent lesions [95]. Other authors found DWI to be at least more accurate than CT in detection of 17 of 24 NSCLC patients with progressive disease after chemotherapy and/or radiotherapy (DWI correct in 24/24, CT in 20/20 with 4 false positives) [96]. Further work confirms that a combination of parameters can improve the detection of recurrence. In a study using DWI MRI on 30 LC patients, local recurrence after lung SBRT was diagnosed in three of 30 without missing any [97]. In practical application, that is, a combination of multi‐parameter MRI and CT may serve to improve the accuracy of response assessment in NSCLC, as Wang et al. have shown after definitive chemoradiotherapy in 150 patients [98].

The observations after chemotherapy and radiation therapy align with effects observed after other therapeutic interventions. In 31 patients after transpulmonary chemoembolization and transarterial chemoperfusion for primary and secondary lung neoplasms, an ADC increase of more than 20.7% indicated volume response with 88% sensitivity and 78% specificity (AUC 0.84) [99]. Similarly, early treatment response after microwave ablation of inoperable lung neoplasms using the ADC value calculated 24 h after the ablation correlated with a higher ADC in responders (cut‐off 1.42 mm^2^/s; 47 patients) [100].

### 4.3 MRI for Radiotherapy Planning of LC

It is not surprising that the above outlined capacities of morphologic and functional MRI for the delineation of tumor extension, differentiation of tumor from surrounding atelectatic lung and post‐obstructive pneumonia, and detection of tissue viability offer interesting options for radiotherapy planning [101]. To remain within the scope of this article, only a few highlights will be mentioned here. For example, a study by Kumar et al. in 26 LC patients explored the principle feasibility of integrating MRI into radiotherapy planning and found a similar interobserver variability in gross target volume (GTV) delineation when using MRI and PET instead of CT and PET [102]. Other authors found that DWI‐derived GTV appeared to be modestly larger than GTV based on PET‐CT [103]. Single‐photon emission CT (SPECT) imaging with technetium‐99 or dual energy CT (DECT) are frequently used to provide functional information prior to RT planning in order to reduce radiation to healthy lungs. As an alternative, Ding et al. have conducted a small pilot study on 10 NSCLC patients integrating functional MRI with Xenon‐129 to reduce radiation exposure in ventilated lung tissue [104]. A meta‐analysis of 19 studies using CT, MRI, SPECT and PET for ventilation‐perfusion imaging of the lung demonstrated that functional imaging including MRI can in fact reduce the functional lung volume that receives 20 Gy by 4.2% [95% CI: 2.3–6.0] and the mean lung dose by 2.2 Gy [95% CI: 1.2–3.3] [105]. During therapy, daily adapted MRI‐based planning can be used to correct for inter‐fraction‐motion, for example, for tumor displacement in collapsing or re‐inflating lung [106, 107]. For motion‐adapted radiotherapy, time‐resolved two‐dimensional (2D + *t*) or three‐dimensional (3D + *t*, a.k.a. 4D‐MRI) imaging opens perspectives for better coverage of moving lung tumors [108]. This approach is bolstered by integration of MRI into radiotherapy units [109]. For example, real‐time, free‐breathing and non‐contrast cine MR‐guided tumor tracking and gating was found to reduce the radiation field without compromising local control in 41 NSCLC patients [110]. Ventilation‐perfusion imaging using Fourier‐MRI has been successfully tested in a 0.35 T MR‐Linac [111].

## 5 Conclusions

The above‐presented selection of multiple research activities yields promising perspectives for lung MRI in the detection, staging, prediction of prognosis, therapy monitoring and follow‐up of LC at variable levels of evidence. High quality randomized controlled trials already exist demonstrating that whole body‐MRI staging pathways have similar accuracy to standard pathways, and reduce the staging time, radiation exposure and cost [65]. Moreover, a position paper by the Fleischner Society acknowledges the greater sensitivity and specificity of multiparametric MRI than CT for characterizing tumor, nodal, and metastatic disease in a single examination and endorses MRI as a means of LC staging [112]. For many other indications, multiple small studies, some of them including 20 patients or less, have produced promising results, but evidence for the robustness in a routine setting and the clinical value still needs to be determined in appropriate clinical trials. Moreover, despite growing technical capabilities, routine clinical adoption of lung MRI is impeded by: limited availability of advanced sequences such as UTE imaging; longer acquisition times than CT; workflow and scheduling constraints in high‐volume clinical settings, particularly emergency departments; reimbursement and cost considerations; and the lack of standardized acquisition and post‐processing protocols across vendors [113]. Addressing these barriers will be essential to enable broader clinical implementation and to facilitate multicenter validation of MRI‐based approaches in LC management [101]. In particular, when high‐quality evidence [65] indicates that MRI offers diagnostic accuracy equivalent to conventional imaging methods without radiation exposure, the ethical principle of nonmaleficence originating from the Hippocratic oath, the “primum nihil nocere,” should be a driving force to intensify the implementation of these new technologies for the sake of reducing patient exposure to detrimental effects, like ionizing radiation or costs in healthcare [114].
